# Early Initiation of and Exclusive Breastfeeding in Large-scale Community-based Programmes in Bolivia and Madagascar

**Published:** 2006-12

**Authors:** Elizabeth Jean Baker, Linda C. Sanei, Nadra Franklin

**Affiliations:** Academy for Educational Development, 1825 Connecticut Avenue, NW, Washington, DC 20009-5721, USA

**Keywords:** Breastfeeding, Infant-feeding practices, Community-based studies, Bolivia, Madagascar

## Abstract

About one-fourth to one-half of all infant deaths in developing countries occur in the first week of life. Immediate breastfeeding within the first hour, followed by early exclusive breastfeeding, improves the health and survival status of newborns. The aim of this study was to demonstrate that breastfeeding practices, crucial to infant health, can be improved at scale in developing countries. During 1999–2003, the LINKAGES Project, funded by the United States Agency for International Development, implemented its community-based model to bring about rapid change in individual behaviours and community norms regarding early and exclusive breastfeeding, at a scale [LINKAGES’ definition of ‘scale’ was adapted from a CORE Group background paper on ‘Scaling-up’ maternal, newborn, and child health services, 11 July 2005] that could achieve significant public-health impact. ‘Scale’ was defined as bringing improved infant-feeding practices to more people over a wider geographic area, more quickly, more equitably, and with sustainability as a goal. During this time, country-specific programmes were designed and implemented in Bolivia and Madagascar, with catchment populations of one million and six million respectively. These country programmes were implemented with multiple local government, private voluntary organizations, and partners of non-governmental organizations (NGOs) through existing health and nutrition activities. Breastfeeding was an entry point to work at all levels of the healthcare system and, within communities, using policy/advocacy and training for healthcare workers, with a particular emphasis on front-line health workers and community members. Harmonized messages and materials, including mass media, were developed and used by partners. Timely initiation of breastfeeding was one indicator measured. Data collected through rapid assessment surveys showed statistically significant increases (p<0.001) in timely initiation of breastfeeding in both the countries. In Bolivia, timely initiation of breastfeeding went from 56% in 2000 to 69% in 2001 and reached 74% by the end of 2003. In Madagascar, the initiation rate went from 34% at baseline in 2000 to 69% in 2001, 76% in 2002, and rose to 78% in 2004. Exclusive breastfeeding during the first month of life was also measured. At baseline in Bolivia, the rate of exclusive breastfeeding for the first month of life was 81% (2000), decreased slightly in 2001, and then increased to 88% by the end of the Project in 2003. In Madagascar, it started high at 86% in 2000, increased during the implementation of the programme, and by 2004, was 91%. These results were achieved quickly and sustained over the course of the intervention.

## INTRODUCTION

Worldwide, about four million babies die annually in the first four weeks of life, i.e. the neonatal period (1). The vast majority of these neonatal deaths occur in the developing world, mainly from preventable causes, and about half take place in the home. The highest numbers of neonatal deaths are observed in the south-central Asian countries, and the highest rates are generally in sub-Saharan Africa (2).

Programmes to reduce neonatal and infant mortality are designed to identify and select those interventions which have the most potential to impact mortality. The promotion of early and exclusive breastfeeding is such an intervention. Early initiation of breastfeeding is characterized as putting the infant to the breast within one hour of birth and is measured using the indicator: timely initiation of breastfeeding. Exclusive breastfeeding means that the infant receives only breastmilk, and the rate of exclusive breastfeeding is the percentage of infants, aged less than six months, who receive only breastmilk and no other solids or liquids, including water, with the exception of drops or syrups consisting of vitamin or mineral supplements or medicines. The benefits of exclusive breastfeeding for infant and child health resulting in reduced rates of morbidity and mortality in early infancy have been extensively reviewed (3–5). The Bellagio Child Survival Study Group highlighted breastfeeding as the lead prevention intervention to improve child health and survival, with estimates that breastfeeding could prevent 13% of all deaths of children aged less than five years (about 1.3 million lives per year) (6). The Collaborative Study Team of World Health Organization (WHO) highlighted the mortality impact using pooled analysis from several countries that documented a greater chance of dying from infectious diseases throughout the first year of life for non-breastfed infants, with the greatest risk for younger infants (7). In the first two months of life, non-breastfed infants had an almost six-fold greater risk of dying from infectious diseases than breastfed infants (7).

Breastfeeding is a foundation practice for appropriate care and feeding of newborn infants (8). The 2003 Guide for Essential Practice for Pregnancy, Childbirth, Postpartum and Newborn Care of WHO identifies early and exclusive breastfeeding as a key component of care (9). The impact of early initiation and exclusive breastfeeding in the first month of life on mortality has recently been documented. A large cohort study undertaken in rural Ghana concluded that 22% of neonatal deaths could be prevented if all infants could be put to the breast within the first hour of birth (10). Additionally, the risk of neonatal mortality was higher among infants who were not exclusively breastfed compared to those who were.

Improved breastfeeding in the neonatal period helps reduce mortality and benefit baby health, growth, and development in the first year and beyond (11). Establishing good breastfeeding practices in the first days after birth is critical to the health of the infant and to breastfeeding success. Initiating breastfeeding is the easiest and most successful when a mother is physically and psychologically prepared for birth and is informed, supported, and confident in her ability to breastfeed and care for the newborn (12). The importance of the community aspects of early breastfeeding support is highlighted in the recent Lancet Neonatal Survival Review that highlights the importance of breastfeeding to neonatal health and cites evidence for a wide range of potential interventions (including early initiation of breastfeeding) (13). It concluded that averting neonatal deaths is possible in settings with high mortality and weak health systems through outreach and family-community care, including health education to improve home-care practices, to create demand for skilled care, and to improve care-seeking (13).

There is also international support and guidance for improving early and exclusive breastfeeding practices. The Global Strategy on Infant and Young Child Feeding (IYCF) (14), co-authored by WHO and United Nations Children's Fund (UNICEF) (2002), is the ‘gold standard’ for guidance to countries that wish to implement improved policies and practices. Community-based interventions to promote and support IYCF are emphasized in the Global Strategy, along with policy measures and improvement of facility-based services. Considerable progress has already been made in the developing world in improving breastfeeding protection, promotion, and support through facility-based services, such as through the Baby Friendly Hospital Initiative (BFHI). Despite this increased focus on community-based approaches and the growing evidence that such approaches can significantly increase optimal practices in diverse settings, few efforts to promote improved IYCF have expanded to a large scale (15). ‘Scale’ is defined as bringing improved infant-feeding practices to more people over a wider geographic area, more quickly, more equitably, and with sustainability as a goal. To expand that experience base, this paper describes the programmes of the LINKAGES Project and its partners to improve, at scale, early initiation and exclusive breastfeeding practices in Bolivia and Madagascar.

## MATERIALS AND METHODS

The LINKAGES Project was a 10-year (1996–2006) global effort funded by the United States Agency for International Development (USAID), managed by the Academy for Educational Development, whose goal was to improve breastfeeding, related complementary feeding, and maternal dietary practices and to support the lactational amenorrhea method of contraception. LINKAGES was mandated to demonstrate changes in breastfeeding behaviours in 2–3 focus countries at a scale to achieve significant public-health impact. During 1999–2004, LINKAGES implemented its community-based model of behaviour change to induce rapid change in individual breastfeeding behaviours and related community norms at scale in countries, including Bolivia and Madagascar. The emphasis of LINKAGES was to demonstrate and document what works at the community level (where most newborn deaths occur) in contrast to smaller-scale earlier breastfeeding efforts which focused primarily on health facilities and hospital-based services in the context of BFHI.

LINKAGES was not designed specifically to target the newborn but because optimal breastfeeding begins at birth, the Project emphasized the neonatal period with timely initiation of breastfeeding (16), one of the core outcome indicators of the Project. Overall, LINKAGES used breastfeeding as an entry point with international and in-country partners by strengthening and expanding the IYCF components within their health and non-health programmes and by using harmonized messages about infant feeding across all programmes. LINKAGES supported a mix of activities for a comprehensive approach. Messages on early initiation of breastfeeding and the importance of early exclusive breastfeeding were used in Bolivia and Madagascar, along with content on this topic in training curricula. They were not limited to the neonatal period but were positioned within the context of the broader set of behaviours required to improve IYCF.

LINKAGES based its evaluation design on criteria that would allow the programme to determine whether impact results shown at scale could plausibly be attributed to the intervention (17). Data for the analysis of these large-scale country programmes came from a series of random-sample, community-level household surveys in Bolivia and Madagascar during which women with infants were interviewed. Baseline surveys were conducted at the start of the intervention, with annual, rapid evaluations carried out to quickly inform the programme of success or lack thereof.

The evaluation design in Madagascar included a comparison control group; this design allows the Project to plausibly state that there is a causal relationship between the intervention and the results. Working with 16 separate non-governmental organizations in Bolivia, the survey was limited to implementation in programme areas only. The lack of a control group in Bolivia means that the results from these three surveys are adequate only to suggest that the intervention has an effect.

In both Bolivia and Madagascar, baseline data were collected in 2000 (Table 1). Follow-up surveys were completed in Bolivia in 2001 and 2003 and in Madagascar in 2001, 2002, and 2004. Respondents were identified based on the age of their youngest child (aged less than 12 months). Survey sampling followed principles of probability sampling. Sampling error was reduced due to the large sample sizes in each country and for each survey round (Table 1).

**Table 1.** Survey background

Country	Survey date	No. (TIBF)	No. (EBF)	Sampling methodology	Survey targets
Bolivia	April 2000	4,327	2,970	Stratified cluster sampling by NGO	Mothers of children younger than 12 months
	October 2001	2,580	1,303	Lot quality assurance sampling by NGO and by health supervision areas	
	May 2003	1,668	834		
Madagascar	February 2000	699	379	Stratified cluster sampling by district	Mothers of children younger than 23 months
	October 2000	195	195	Purposeful stratified cluster sampling by district (most active communes only)	
	October 2001	199	199		
	October 2002	180	180		
	November 2004	320	320		

EBF=Exclusive breastfeeding;

NGO=Non-governmental organization;

TIBF=Timely initiation of breastfeeding

Multi-state cluster sampling was used in Madagascar so that there was an equal probability of selection for clusters within clusters; every cluster had a non-zero probability of selection, and a regional effect could be detected (18). This design is based on the EPI methodology (WHO's Expanded Programme on Immunization), a two-stage cluster sampling with probability proportional to size. Using a control group for comparison, we attempted to rule out alternative hypotheses to explain behaviour change other than the programme intervention and, thus, make a plausible argument for causality.

A stratified cluster-sample approach was used in Bolivia for ensuring that each NGO partner would have reliable data at the NGO level and that LINKAGES would have valid programme-level data. These three-year trend data in Bolivia follow the course of the intervention and are adequate to suggest a programme-intervention effect on change in infant-feeding behaviour during the neonatal period.

In each country, the sample population was stratified by age so that an adequate sample size of the specific age-groups of 0–5 and 5–11 months would be generated. Where the sample was stratified by NGO, the data were weighted during analysis so that the probability of selection for each individual across NGOs would be equal.

Each survey was implemented following the standard procedures for assuring verbal consent to participate in the survey, and each respondent was advised that their answers would remain anonymous in the data entry and reporting process. Data were collected on breastfeeding and related infant-feeding behaviours that were supported by the Project interventions; all infant-feeding indicators, including timely initiation of breastfeeding and exclusive breastfeeding, adhere to the definitions and calculations advocated by WHO (19). These same indicators are similarly collected in the Demographic and Health Surveys and in the UNICEF's Multiple Indicator Cluster Survey. Sociodemographic data of women and programme exposure data were also collected.

### Bolivia programme intervention

In 1997, LINKAGES began work in Bolivia where 28% of children, aged less than three years, suffer from chronic malnutrition. The high rates of infection and malnutrition and the estimated 4,500 annual infant deaths are attributable in part to sub-optimal breastfeeding practices. To reduce neonatal morbidity and mortality, LINKAGES focused its programme primarily on: (a) increasing the rate of timely initiation of breastfeeding by 10% and (b) increasing the rate of exclusive breastfeeding by 15%.

To realize coverage at scale, LINKAGES worked with PROCOSI, an umbrella organization of international private voluntary organizations (PVOs) and local NGOs. PROCOSI was supported by USAID Bolivia, and the work of its member organizations was targeted to rural, under-served, sparsely-populated, and poverty-stricken areas. In total, 16 partner NGOs that supported child survival, reproductive health, integrated health, food security, and other health-related interventions committed to work with LINKAGES to increase timely initiation of breastfeeding and rate of exclusive breastfeeding within their respective catchment areas. The Project activities were carried out by community health workers already employed by the partner NGOs of the PROCOSI network. By 2003, the Project catchment population was one million, covering eight of nine departments in Bolivia, three eco-regions, 155 municipalities, and 2,389 communities.

To increase timely initiation of breastfeeding and early rate of exclusive breastfeeding, regional behaviour-change workshops were conducted to analyze factors that interfere with optimal initiation and maintenance behaviours in each eco-region so that approaches, messages, and materials could be custom-tailored to the local populations in the local dialects. From this analysis, a participatory approach among all NGOs was used for developing a 12-panel cloth flipchart, six laminated counselling cards, manuals for community health workers, an educational video, radio spots and testimonials, a radio call-in programme, radio drama series, calendars, etc. in the target communities.

The programme was implemented in phases. Phase I included formative research, a literature review, and needs and resource assessment. Phase II focused on advocacy at the national level which was later targeted at regional and district levels in the NGO coverage areas. Phase III constituted the materials-development phase and Phase IV focused on training. Training was a key aspect of the programme targeted at NGO staff, Ministry of Health counterparts, doctors, nurses, auxiliary nurses, and community health workers. Both training and behaviour change-communication activities emphasized a range of infant and young child-feeding behaviours which included, but were not limited to, timely initiation of breastfeeding and early exclusive breastfeeding. Several types of training and curricula were developed (such as behaviour change communication and mother-to-mother support) and conducted at various levels that included community-level volunteers and health workers. The Project target was to train 80% of the community personnel of the PROCOSI partner NGOs, with the goal that of those trained, 80% could correctly identify at least five basic IYCF and LAM messages.

Nearly 100% of the training targets established were achieved during the implementation of the programme. In all, 1,700 community health workers, 200 doctors and nurses, and 350 auxiliary nurses were trained, and 1,600 community health workers received refresher training. Knowledge of the community health workers about the programme's key messages and technical content areas, skills in counselling and negotiation techniques, and in the use of visuals, to enable negotiation and counselling during home-visits, educational talks in the community, facilitation of mother-to-mother support-group, local health-fair participation, and health-facility referrals of appropriate cases, were strengthened.

### Madagascar programme intervention

In Madagascar, the LINKAGES’ programme interventions evolved and expanded through several phases. During 1997–1999, the initial phase centred on national policy activities through the involvement of governmental, non-governmental, and donor organizations, broadly focused on nutrition. The result was the establishment of a national inter-sectoral nutrition group that oversaw harmonization of nutrition messages and materials across group members, development of nutrition guidelines, including those on early initiation and exclusive breastfeeding, and agreement on common protocols for the implementation of the programme by all partners. This phase created a favourable environment for the extensive programme-implementation activities that followed.

In 1999, LINKAGES moved to a second phase of district and community activities as a partner with the Ministry of Health and others in an integrated child-survival and reproductive health bilateral project funded by USAID, Madagascar. Breastfeeding was a ‘lead’ intervention in a broader set of nutrition behaviours (called essential nutrition actions), that along with other child-survival and reproductive health interventions formed the core of the bilateral project. By 2004, the Project covered over six million people.

With its partners, emphasis of LINKAGES was on key infant-feeding indicators, including timely initiation of breastfeeding and exclusive breastfeeding. Messages on early initiation were based on formative research which identified obstacles to optimal behaviours. For example, research suggested that the decision to feed colostrum was not always made by the mother, since it was frequently the midwife or traditional birth attendant who decided when the mother started breastfeeding. Available data suggested that feeding colostrum was already fairly well-accepted by Malagasy mothers when the programme began and primarily needed to be reinforced.

The behaviour change communication (BCC) approach of LINKAGES was on priority messages of ‘small do-able actions’, i.e. mother should allow infant to breastfeed on demand (as often as infant wants). These messages were transmitted through behaviour-change materials, such as health booklets and health cards, developed for mothers and families, and through materials for health workers which included job aids and counselling cards. To enhance scale and reach, multiple channels were used in this BCC effort aimed at ‘saturating’ the country and included interpersonal communications, community mobilization (village theatre and festivals, and other popular community events), and mass media (newspapers, radio, and television). Also effective was a ‘high-profile’ endorsement by a popular female Malagasy rock singer who was breastfeeding her own baby.

Behaviour change was emphasized at all levels, from the national focus on policy, through training of health workers at the district level, to support for women's groups at the community level to educate about and reinforce practice of these behaviours. In addition to messages, various points of contact with mothers and families (antenatal care, delivery, postnatal visit, immunization, well baby visit, and sick child visit) were all used for educating and motivating families. National-level advocacy efforts were undertaken aimed at key policy and decision-makers. Pre-service training was provided in essential nutrition actions to medical and nursing schools.

To reach increased scale through expanded geographic coverage, strategic partnerships were formed with existing Ministry of Health service-delivery sites and those of PVOs and local NGOs. Since the programme was primarily carried out under the auspices of the Ministry of Health, the majority of the community health workers were employees of the Ministry of Health. However, volunteers also carried out some community-based activities. The role of LINKAGES in the partnership was to offer training for health workers, community volunteers, and members of women's groups which emphasized ‘negotiation skills’ for establishing a dialogue with a mother, rather than just passing didactic message content or dispensing advice.

## RESULTS

Analyzing the survey data, LINKAGES demonstrated that the intervention had the intended positive effects on neonatal infant-feeding behaviours at scale in both Bolivia and Madagascar.

**Fig. 1.**
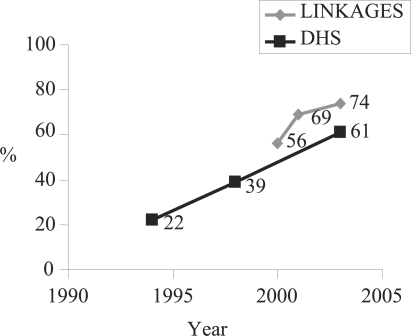
Timely initiation of breastfeeding, LINKAGES Bolivia, and DHS Bolivia

Rates of timely initiation of breastfeeding and exclusive breastfeeding within the preceding 24 hours, by infants aged less than one month, were calculated for each survey in each country. Of all women with infants aged less than 12 months in Bolivia, the rate of timely initiation of breastfeeding increased from 56% in 2000 to 69% in 2001 and to 74% in 2003 by the end of the Project intervention. The increase from baseline to the end of the Project was statistically significant (p<0.001). These results in the catchment communities are similar to the national-level secular trend increases reported in the Demographic and Health Survey (DHS), although the slope of the change in the programme communities is higher between the baseline and the final survey than for the corresponding time period in the DHS (Fig. 1).

DHS=Demographic and Health Survey

**Fig. 2.**
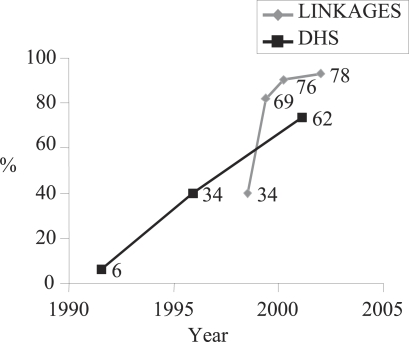
Timely initiation of breastfeeding and early exclusive breastfeeding. LINKAGES Bolivia

Exclusive breastfeeding during the first month followed a similar pattern of increase, although baselines for the exclusive breastfeeding rate were higher than for timely initiation (Fig. 2). At baseline, exclusive breastfeeding of infants during their first month was 81%, decreased slightly to 75% in 2001 and then increased to 88% by the end of the Project in 2003; this increase from 2000 to 2003 was statistically significant (p<0.001). Rates of exclusive breastfeeding remained higher than rates of breastfeeding initiation at each point in time.

DHS=Demographic and Health Survey

Over the course of the programme, community members were exposed to the same message from multiple sources. Messages on timely initiation of breastfeeding by the end of the intervention were heard primarily from health workers, and the community health promoters supported through the intervention (Table 2). Exposure to messages on the early initiation of breastfeeding through home-visits by health promoters and mother-to-mother support groups remained low over the course of the intervention. Seven of the 16 partner NGOs participated in mother-to-mother support group training, and this intervention was, thus, limited in application over the catchment area. Messages on exclusive breastfeeding emphasized exclusive breastfeeding for the full six months and not on individual month intervals.

**Table 2.** Percentage distribution of source of message for women who initiated breastfeeding within one hour of birth

Message exposure	Baseline (2000)	Endline (2003)
Health worker	61**	69**
Community health promoter	9**	31*
Family member	17**	12*
Radio	4	10
Home-visit (health-education promoter)	3	7
Mother-to-mother support Group	2	6
Neighbour/friend	4**	4**
Do not know	5*	4**
Other	8**	1

The sample is of women who were identified through the survey data as having initiated breastfeeding within the first hour. This sub-sample was analyzed to determine where they heard messages on early initiation of breastfeeding

**p<0.001,

*p<0.01

**Fig. 3.**
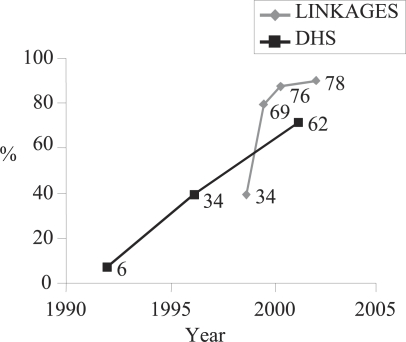
Timely initiation of breastfeeding, LINKAGES Madagascar and DHS Madagascar

**Fig. 4.**
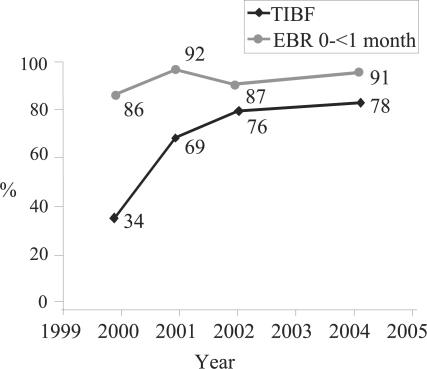
Timely initiation of breastfeeding and early exclusive breastfeeding, LINKAGES Madagascar

In Madagascar, the rate of timely initiation of breastfeeding was low at baseline (34%); this was the same as the national rate recorded in the DHS 1988 (Fig. 3). Over time, timely initiation of breastfeeding increased to 69%, 76%, and 78% (2004) (Fig. 4). This increase in the mean rate of timely initiation was higher than that observed in national-level DHS data despite that rates for the programme area were the same at the start of the intervention as the national rate was two years prior to the start of the intervention. A cost-effectiveness analysis was carried out to measure actual behaviour change per dollar of investment in the intervention (20). Using the measure “cost-effectiveness per new immediate initiation of breastfeeding acceptor”, the cost of every new timely breastfeeding initiation acceptor was only US$ 2.33, suggesting that this was a cost-effective large-scale intervention.

DHS=Demographic and Health Survey

EBR=Exclusive breastfeeding
TIBF=Timely initiation of breastfeeding

Similar to experiences in Bolivia, the prevalence of exclusive breastfeeding during the first month was already high at the start of the intervention and remained consistently high during the implementation of the programme. While annual rates went both up and down, by 2004, exclusive breastfeeding was higher (91% in 2004) at the most recent data-collection point than at baseline (86% in 2000) and remained higher than rates of breastfeeding initiation.

While messages were conveyed through multiple sources in Madagascar as they were in Bolivia, survey data on messages of breastfeeding-initiation were not collected. As in Bolivia, exclusive breastfeeding messages were for the full six months and were not targeted at specific age intervals. Thus, the relationship between exclusive breastfeeding during the first month and the sources of the messages cannot be determined.

## DISCUSSION

Although LINKAGES was not designed as a neonatal project, optimal breastfeeding begins at birth and the infant-feeding practices established in the early days after delivery provide an important foundation for good infant feeding throughout the life of the infant. LINKAGES, therefore, adopted the ‘gold standard’ of timely initiation of breastfeeding as one of its key indicators, along with exclusive breastfeeding to six months. The LINKAGES programme approach emphasized changing community support and norms around breastfeeding, acknowledging that a large percentage of births still take place in the home. The BCC efforts of the programme emphasized small actions that can be taken by mothers, birth attendants, and health workers to improve the breastfeeding practices. Messages delivered through multiple channels gave the same guidance about a specific, simple action to be taken, such as “put the baby to the breast within the first hour after birth” rather than those on the general benefits of breastfeeding.

Timely initiation of breastfeeding was relatively high at 56% in 2000 when LINKAGES began work in Bolivia. The significant increase to 74% by 2003 suggests that this is a relatively easy behaviour to change at scale when communities and health providers are sensitized through training and BCC to its importance to infant health. While timely initiation of breastfeeding was at a lower level in Madagascar in 2000, the pattern of increase was similar to that of Bolivia, topping out in 2004 at 78%. The fact that timely initiation of breastfeeding is a ‘one-time’ activity may also make it more susceptible to change than the repeated actions necessary to carry out exclusive breastfeeding up to the age of six months. In Madagascar, cost-effectiveness analysis demonstrated that carrying out this large-scale intervention yielded behaviour-change results economically.

Since breastfeeding is a cultural norm, and it is well-accepted that breastfeeding benefits infants in both cultures, the challenge was to ‘move’ the current breastfeeding practices closer to optimal. This meant an emphasis on delaying introduction of any additional liquids (even water) and foods, ideally to six months. The behavioural emphasis of LINKAGES was to target this information to mothers, caretakers, and health workers who influence infant-feeding behaviours. This was done in Bolivia in the context of ongoing child-survival or reproductive health programmes of the PROCOSI partner PVOs, while in Madagascar the implementation was through the Ministry of Health delivery system, especially MCH services.

Because of the proven public-health impact of improved breastfeeding on infant morbidity and mortality, the objective of LINKAGES from the outset was to support behaviour change for IYCF to a large population in a short time, hence the emphasis on scale in both Bolivia and Madagascar. When the term ‘scale’ is used with regard to health programmes, it is commonly understood to mean a large number of beneficiaries covered by some combination of interventions. LINKAGES adopted similar use for its country programmes, where ‘scale’ meant delivery of improved infant feeding provided to a large number of beneficiaries (one million or more), with expanding geographic coverage, national-level policy/advocacy, and intent to address sustainability in the programme design. In both the countries, LINKAGES scaled up not only infant-feeding technical interventions but also the processes needed to improve the status of those behaviours. Programme data of LINKAGES on its key infant-feeding indicators from Madagascar and Bolivia document such an impact at scale.

The experience of LINKAGES in Bolivia and Madagascar suggests that large-scale programmes with a strong community behaviour change focus can improve immediate breastfeeding within the first hour, and exclusive breastfeeding, with significant potential to improve the health and survival of newborns.

Implications for large-scale community-level behaviour change include the following:

- Think ‘big’, develop a shared vision of scale with stakeholders, and make the commitment to scale at the very start of the assessment phase. However, be aware that this collaborative process is both labour- and time-intensive.
- Realize that there are different ways to go to scale. The most successful strategy is likely to be one that employs various approaches, is situational and ‘opportunistic’, and is based on assessment of the readiness, potential, and reach of potential local partners (such as the Ministry of Health, PVOs, NGOs, other local organizations, donors). The country context and local environment help determine the design of the scale process.
- During the country programme design phase, cultivate a blending of ideas, opinions, and perspectives—this yields more productivity and a better result. Our planning and assessment phase was completed using a team approach and engaging multi-level stakeholders.
- Sensitize key in-country players from the national level to the local community leaders to your ideas, through a marketing approach and using various tools and approaches. For example, policy and advocacy tools, such as PROFILES, helped create awareness and sensitivity to the biological and functional outcomes of optimal (or suboptimal) nutrition patterns.
- Identify enough local partners to gain adequate geographic coverage; this is crucial to going to scale. Strategically-selected partners are essential to reach a ‘tipping point’ for changes in desired behaviours and, ultimately, social norms. Keep partners motivated with benefits, such as providing refresher training and by sharing evaluation results documenting programme success.
- Ensure adequate programme staffing to support the scale of intervention activities. Partners provide human and other resources needed to get messages out and create community ‘noise’ about the programme.
- Identify a set of small, doable actions, such as ‘initiate breastfeeding within one hour of birth’, that lead to the use of consistent messages across the entire catchment area, by multiple stakeholders and that are all tied to standard intervention indicators. These messages should be adopted and diffused by all partners.
- Simultaneously support a mix of activities for a comprehensive approach with stakeholders, combining short-term and longer-term interventions. The specific mix of activities will be dependent on the local situation. LINKAGES began with an emphasis on community interventions intended to bring about results in the short term, such as community-based training of health workers, group and individual counselling, media, and mothers’ support clubs. Longer-term interventions, intended to consolidate and sustain results, such as pre-service training for medical and nursing staff, were introduced later in the Project.
- Saturate the catchment population with repeated messages from multiple sources for a positive effect on behaviour.
- Compromise and be flexible in programme roll-out, allowing for innovation and adaptation to the local situation. LINKAGES streamlined its BCC approach to gain its partners’ agreement, participation, and buy-in for the approach, and in Madagascar, trimmed the training cycle to three-day training workshops. In short, the implementation can be modified as necessary while still maintaining the technical integrity of the approach.

We do caution that scale may not be appropriate for, or applicable to, all health interventions. In particular, interventions that require testing or are highly politicized may not be well-suited to going quickly to scale. However, well-established, accepted, and scientifically-based behaviours, such as timely initiation of breastfeeding and early exclusive breastfeeding, are appropriate for scale.

## ACKNOWLEDGEMENTS

Funding for the programmes described in this paper was provided by the United States Agency for International Development (USAID) to the LINKAGES Project (1996–2006), managed by the Academy for Education Development (AED) under the terms of Cooperative Agreement No. HRN-A-00-97-00007-00. The opinions expressed in this paper are those of the authors and do not necessarily reflect the views of USAID or AED, or its officers and staff. Additional information on the LINKAGES Project can be found at www.linkagesproject.org.

The authors are grateful to Elizabeth Weinstein for her research and editorial assistance and to Jay Ross for his technical review of multiple versions of the paper. The authors also thank their colleagues Luann Martin, Margaret Parlato, and Renata Seidel for their thoughtful review of the final version of the paper. Thanks are extended to LINKAGES staff responsible for the management, implementation, and evaluation of the country programmes—Agnès Guyon, Michael Hainsworth, Victoria Quinn, Zo Rambeloson, Voahirana Ravelojaona, Priscilla Ravonimanantsoa, Maryanne Stone-Jiménez, and Albina Torres. Special acknowledgments are extended to the in-country partners—governments, non-governmental organizations, communities, the PROCOSI network (Bolivia), and USAID Missions.
